# Development of a Potent and Protective Germline-Like Antibody Lineage Against Zika Virus in a Convalescent Human

**DOI:** 10.3389/fimmu.2019.02424

**Published:** 2019-10-24

**Authors:** Fei Gao, Xiaohe Lin, Linling He, Ruoke Wang, Han Wang, Xuanling Shi, Fuchun Zhang, Chibiao Yin, Linqi Zhang, Jiang Zhu, Lei Yu

**Affiliations:** ^1^Department of Basic Medical Sciences, Comprehensive AIDS Research Center, Beijing Advanced Innovation Center for Structural Biology, School of Medicine, Tsinghua University, Beijing, China; ^2^Department of Integrative Structural and Computational Biology, Department of Immunology and Microbiology, The Scripps Research Institute, La Jolla, CA, United States; ^3^Guangzhou Eighth People's Hospital, Guangzhou Medical University, Guangzhou, China

**Keywords:** Zika virus infection, Guillain–Barré syndrome, microcephaly, neutralizing antibody, antibody repertoire, next-generation sequencing

## Abstract

Zika virus (ZIKV) specific neutralizing antibodies hold great promise for antibody-based interventions and vaccine design against ZIKV infection. However, their development in infected patients remains unclear. Here, we applied next-generation sequencing (NGS) to probe the dynamic development of a potent and protective ZIKV E DIII-specific antibody ZK2B10 isolated from a ZIKV convalescent individual. The unbiased repertoire analysis showed dramatic changes in the usage of antibody variable region germline genes. However, lineage tracing of ZK2B10 revealed limited somatic hypermutation and transient expansion during the 12 months following the onset of symptoms. The NGS-derived, germline-like ZK2B10 somatic variants neutralized ZIKV potently and protected mice from lethal challenge of ZIKV without detectable cross-reactivity with Dengue virus (DENV). Site-directed mutagenesis identified two residues within the λ chain, N31 and S91, that are essential to the functional maturation of ZK2B10. The repertoire and lineage features unveiled here will help elucidate the developmental process and protective potential of E DIII-directed antibodies against ZIKV infection.

## Introduction

Zika virus (ZIKV), a member of the *Flavivirus genus* of the *Flaviviridae* family, is an emerging mosquito-borne pathogen. ZIKV is closely related to other flaviviruses such as dengue (DENV 1, 2, 3, and 4), yellow fever (YFV), West Nile (WNV), Japanese encephalitis (JEV), and tick-borne encephalitis (TBEV) viruses (1). Since ZIKV was first identified in 1947 among rhesus macaques in the Zika forest of Uganda, its new variants have become increasingly prevalent and have adapted to the human population as recent outbreaks spread across the Americas, Caribbean, and Southeast Asia (2–5). At the peak of the 2016 outbreak, several incidents of imported ZIKV infection were identified in mainland China (6). In contrast to previous epidemics, the recent ZIKV outbreak has been associated with severe neurological complications such as Guillain–Barré syndrome in adults and microcephaly in fetuses and newborns (7–10). Currently, no ZIKV-specific therapeutics or vaccines are available. The high prevalence of the vectors and the continuing evolution of viral species have raised serious concerns about public health and ZIKV-related disease control (11).

The surface envelope glycoprotein (E) of flaviviruses mediates entry and presents a potential target for neutralizing antibodies. Large numbers of E-targeting monoclonal antibodies (mAbs) have been identified with potent neutralizing activity and epitope specificity (12–29). Previously, we isolated and characterized a panel of E-targeting mAbs from plasma and memory B cells from sequential blood samples of a DENV-naïve ZIKV-infected convalescent patient (Pt1) who acquired ZIKV infection in Venezuela during the 2016 outbreak and then returned to China (6, 24). Among these mAbs, ZK2B10 showed the highest neutralizing potency against ZIKV without any detectable reactivity with DENV 1 or 2 (24). ZK2B10 also demonstrated remarkable prophylactic and therapeutic activities against lethal challenge in the mouse models of ZIKV infection and microcephaly (30). Crystal structure and cryo-EM analysis revealed that ZK2B10 recognizes the lateral ridge of E DIII and blocks infection by inhibiting membrane fusion after cellular attachment (31). Since ZK2B10 may serve as a promising candidate for antibody-based interventions, the ontogeny of ZK2B10 could provide insight into the protective antibody response during ZIKV infection in humans and inform rational vaccine design. Furthermore, diverse vaccine candidates have demonstrated their ability to protect against ZIKV challenge in mice or nonhuman primates (NHPs) and have been evaluated in preclinical and clinical studies (16, 32, 33). It is therefore imperative to investigate the dynamics and characteristics of the antibody repertoire during ZIKV infection longitudinally, which will shed light on the molecular requirements necessary for the development of an effective ZIKV vaccine.

In this study, we applied long-read next-generation sequencing (NGS) and an unbiased repertoire capture method to longitudinally analyze the B cell repertoire of Pt1 from the early acute phase to the late convalescent phase (34). We obtained tens of millions of antibody sequences from a total of seven sequential time points including Day 4, Day 15, Month 2, Month 3, Month 6, Month 10 and Month 12 after the onset of symptoms. We first performed NGS analysis of the antibody repertoire with a focus on germline gene usage, CDR3 loop length, and degree of somatic hypermutation (SHM). Our data revealed that the antibody repertoire profile during ZIKV infection consisted of diverse germline gene usage combined with a steady distribution of CDR3 loops, in contrast to chronic HIV-1 infection, which often exhibits unusual repertoire profiles characteristic of high degree of SHM, skewed germline gene usage, and long HCDR3 loops (34, 35). The emergence of germline-like antibodies was observed at Day 15 after the onset of symptoms. We then traced the antibody lineage of ZK2B10 within the NGS-derived repertoire and investigated its maturation pathway. Our results show that ZK2B10 was generated with relatively low titers along with other germline-like antibodies at Day 15. Somatic variants of ZK2B10 were synthesized for functional characterization both *in vitro* and *in vivo*. Germline-like ZK2B10 heavy chain variants demonstrated strong neutralizing activity and protection against lethal ZIKV challenge in a mouse model. Of note, two substitutions occurred at positions N31 on LCDR1 and S91 on LCDR3 of λ-light chain were found to be critical for the functional maturation of ZK2B10. In summary, our repertoire and lineage analyses elucidated the maturation pathway of a potently neutralizing antibody, ZK2B10, and suggested that germline-like antibodies may play an important role in protective immunity against ZIKV infection.

## Materials and Methods

### Donor and PBMCs Samples

The blood samples were donated by a 28-year-old Chinese ZIKV convalescent male patient (Pt1) who traveled from Venezuela to the southern metropolitan city Guangzhou, China, in February, 2016 (6). During his hospitalization and follow-up visits, a total of 7 sequential blood samples were collected at Day 4, Day 15, Month 2, Month 3, Month 6, Month 10, and Month 12 after the onset of symptoms. Samples were separated into plasma and peripheral blood mononuclear cells (PBMCs) by centrifugation through a Ficoll-Hypaque gradient (GE Healthcare). PBMCs were cryopreserved in freezing media and stored in liquid nitrogen until further analysis by antibody repertoire sequencing.

### Sample Preparation Using 5′-RACE PCR

An improved version of the rapid amplification of cDNA 5′-ends (5′-RACE) polymerase chain reaction (PCR) protocol for sample preparation was reported in a recent study (34, 36). Here, total RNA was extracted from 1~5 million PBMCs into 30 ml of water with RNeasy Mini Kits (Qiagen, Valencia, CA). For unbiased repertoire analysis, 5′-RACE was performed with SMARTer RACE cDNA Amplification Kit (Clontech, Mountain View, CA). For ZK2B10 gene-specific lineage analysis, reverse transcription (RT) was performed with SuperScript III (Life Technologies) and oligo (dT). In both cases, the cDNA was purified and eluted in 20 μl of elution buffer (NucleoSpin PCR Clean-up Kit, Clontech). The immunoglobulin PCRs were set up with Platinum Taq High-Fidelity DNA Polymerase (Life Technologies, Carlsbad, CA) in a total volume of 50 μl, with 5 μl of cDNA as template, 1 μl of 5′-RACE primer or gene-specific forward primers, and 1 μl of 10 μM reverse primer. To facilitate deep sequencing on the Ion GeneStudio S5 system, the forward primers (both 5′-RACE and gene-specific) contained a P1 adaptor, while the reverse primer contained an A adaptor and an Ion Xpress^TM^ barcode (Life Technologies) to differentiate the libraries from various time points. A total of 25 cycles of PCRs were performed and the PCR products (~600 bp for 5′-RACR PCR or ~500 bp for gene-specific PCR) were gel purified (Qiagen, Valencia, CA). A degenerate primer (SAGGTGCAGCTGGTGCAGTCTGG) was used as the forward gene-specific primer to cover potential variations at the 5′-end of ZK2B10 transcripts.

### Next-Generation Sequencing (NGS) and Antibodyomics Analysis

Antibody NGS has been adapted to the Ion GeneStudio S5 system (35). Briefly, the antibody heavy and light (κ and λ) chain libraries were quantitated using Qubit^®^ 2.0 Fluorometer with Qubit^®^ dsDNA HS Assay Kits. Equal amounts of the heavy chain libraries from various time points were mixed and loaded onto an Ion 530 chip to increase the sequencing depth and to eliminate run-to-run variation. The κ and λ chain libraries at each time point were mixed at a ratio of 1:1 prior to library pooling and chip loading. Template preparation and (Ion 530) chip loading was performed on the Ion Chef system using Ion 530 Ext Kits, followed by S5 sequencing with the default settings. Raw data was processed without 3′-end trimming in base calling to extend the read length. The human *Antibodyomics* pipeline version 1.0 (34, 36, 37) has been modified to improve data accuracy and computational efficiency (35). This new *Antibodyomics* pipeline was used to process and annotate Pt1 antibody NGS data for repertoire profiling and lineage tracing. The distributions of germline genes, germline divergence or degree of SHM, and CDR3 loop length derived from antibody NGS data as general repertoire profiles. The two-dimensional (2D) divergence/identity plots were constructed to visualize ZIKV-specific antibody lineages in the context of Pt1 antibody repertoire. A CDR3 identity of 95% was used as the cutoff for identifying sequences evolutionarily related to a reference antibody (shown as magenta dots on the 2D plots). The hierarchical clustering method was used to divide CDR3-defined somatic variants into groups based on an overall identity cutoff of 98% as previously described (34). In addition to the dominant sequences, a consensus or a manually selected sequence was used as the group representative for antibody synthesis and functional characterization. ZK2B10 were initially isolated from PBMCs of Pt1 as we previously reported (24).

### Human Monoclonal Antibody (mAb) Clones Construction, Expression, and Purification

All of the synthetic variable region genes of antibody heavy chain (V_H_) and light chain (V_K/L_) were analyzed using the IMGT/V-Quest server (http://www.imgt.org/IMGTindex/V-QUEST.php). They were cloned into the backbone of antibody expression vectors containing the constant regions of human IgG1 as previously described (38). To produce full-length human mAbs, the recombinant clone was paired with the complementary chain of wild-type (WT) ZK2B10. The heavy and light chain expression plasmids were transiently co-transfected into HEK 293T cells for the production of full-length human IgGs, which were purified from the supernatant by affinity chromatography using protein A agarose (Thermo Scientific). The IgG concentration was determined using the BCA Protein Assay Kit (Thermo Scientific). We included previously reported MERS-CoV-specific mAb MERS-4 (38) for comparative analysis.

### ZIKV E and ZIKV E DIII Protein and ELISA

The gene of either E protein or E DIII protein (residues 301-403) of ZIKV (GZ01, KU820898) without tag was cloned into pET28a vectors (Novagen) and expressed by IPTG-induction in BL21 (RIL) bacterial cells. The isolated inclusion bodies were solubilized and re-folded as reported (39). In the enzyme-linked immunosorbent assay (ELISA), the E proteins and E DIII proteins were captured separately onto ELISA plates overnight at 4°C. Each tested mAb was serially diluted and applied to the ZIKV E and E DIII protein-captured ELISA plates. Binding activities were detected using anti–human IgG labeled with HRP and TMB substrate.

### Antibody Neutralization Assays

All ZIKV GZ01 (KU820898), ZIKV MR766 (AY632535), and DENV2 43 (AF204178) viruses were grown in C6/36 Aedes Albopictus cells and titrated on Vero cells before use. For neutralization assay, serial dilutions of mAbs were mixed with virus at 4°C for 1 h before being applied to Vero cells in the 6-well culture plates. After 1–2 h of infection, the antibody-virus mixture was aspirated and Vero cells were washed with PBS and overlaid with DMEM containing 2% heat-inactivated FBS and 1% SeaPlaque Agarose (Lonza, 50501). After 4–6 days, plaques were stained by 1% crystal violet and counted manually.

### Antibody Prophylactic Potential Analysis in AG6 Mice

C57BL/6 mice deficient in interferon (IFN) α, -β, and -γ receptors (AG6 mice) were kindly provided by the Institute Pasteur of Shanghai, Chinese Academy of Sciences (IPS). The mice were bred and maintained in a pathogen-free animal facility. Groups of 4 sex-matched, 4- to 6-week-old AG6 mice were used for the animal studies. In prophylaxis assays, 300 μg of each tested mAb or isotype control (MERS-4) was administered via the i.p. route. The following day, the animals were challenged with 10^4^ PFUs of ZIKV (GZ01 strain) via i.p. injection. Survival was monitored for up to 14 days post challenge. At days 5 and 12 after challenge, whole blood was collected from each animal for ZIKV viral load measurement.

### Quantitative Measurement of Viral Loads by TaqMan qpcr

Whole blood (10 μL) was collected in an RNase free Eppendorf tube containing lysis buffer (QIAGEN) and stored at −80°C until use. Total RNA was extracted using RNeasy Mini Kits (74106, QIAGEN) and reverse-transcribed into cDNA using iScript cDNA Synthesis Kits (170-8890, Bio-Rad). Viral RNA copies were quantified through TaqMan qPCR amplification of ZIKV (GZ01) envelope gene. Measurements were expressed as log_10_ viral RNA copies per millimeter calculated against a standard curve. Sequences for primers and probes were as follows: ZIKV-F CCGCTGCCCAACACAAG, ZIKV-R CCACTAACGTTCTTTTGCAGACAT, ZIKV-probe AGCCTACCTTGACAAGCARTCAGACACTCAA (5′FAM, 3′TAMRA).

### Multiple Sequence Alignment and Structural Analysis

Multiple sequence alignment (MSA) was calculated using BioEdit ClustalW. The crystal structure of ZIKV E DIII-ZK2B10 Fab complex has been determined and analyzed here to identify the ‘hotspot' residues critical to ZK2B10 lineage development (31). For the intermolecular interactions shown in **Figure 5**, 4 Å was used as the maximal cut-off distance for hydrogen bonds. Illustrations of structural models were prepared using PyMOL Molecular Graphics System 1.5.0.4.

### Statistical Methods

All data were analyzed using Prism6 software (GraphPad). The half-maximal effective concentrations (EC_50_) were calculated using the dose-response stimulation model. The IC_50_ value for each mAb was calculated using the dose-response inhibition model. For experiments involving AG6 mice, four animals were included in each assessment group to ensure equal representation and consistency of the data obtained. Statistical analysis was performed using Student's unpaired t test. Data were presented as mean ± SEM. ^*^p < 0.05; ^**^p < 0.01; and ^***^p < 0.001.

### Ethics Statement

The human study was approved by the Ethical Committee of the Guangzhou Eighth People's Hospital, Guangzhou Medical University. The research was conducted in strict accordance with the Chinese government rules and regulations for the protection of human subjects. The study subjects provided the written informed consents for research use of their blood samples. All procedures with animals were undertaken according to Experimental Animal Welfare and Ethics Committee of Tsinghua University. All experiments were performed under the guidelines of the Experimental Animal Welfare and Ethics Committee of Tsinghua University (16-ZLQ9).

## Results

### Dynamic B Cell Repertoire Response Throughout ZIKV Infection

**Figure 1:**
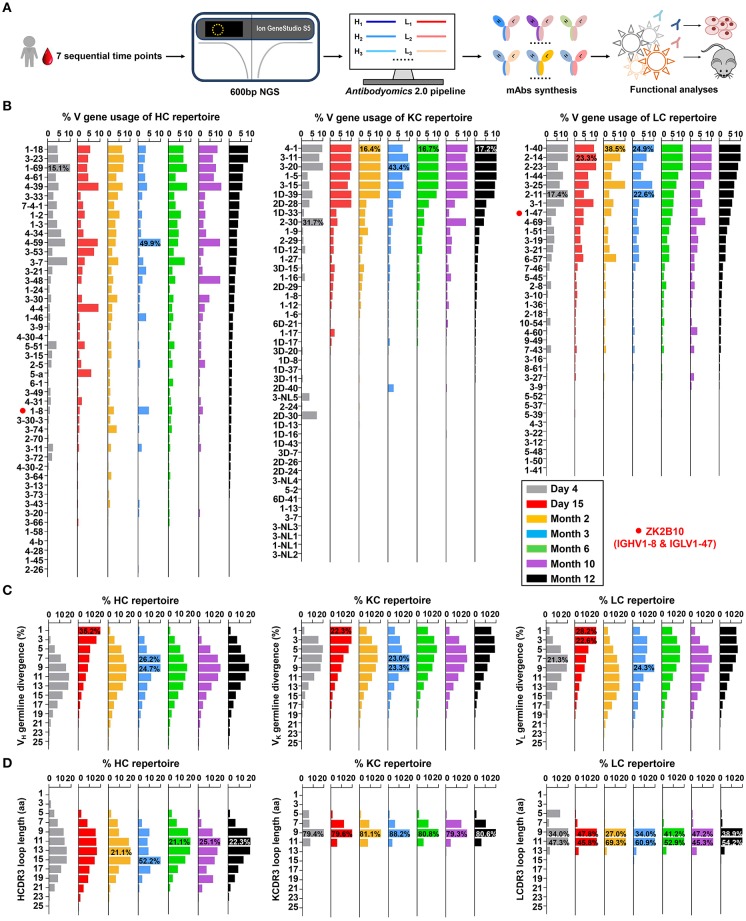
Unbiased antibody repertoire profiles of donor Pt1 across ZIKV infection. **(A)** Schematic view of unbiased antibody repertoire analysis and ZK2B10 lineage tracing. PBMC samples from Pt1 were collected at 7 sequential time points after the onset of symptoms. 5′-RACE PCR was used to prepare antibody chain libraries for long-read (600 bp) next-generation sequencing (NGS) on the Ion GeneStudio S5 platform. The *Antibodyomics* 2.0 pipeline was used to process the NGS data for antibody repertoire profiling, while CDR3-based identification was used for ZK2B10 lineage tracing. Representative somatic variants were synthesized for functional characterization. **(B–D)** Distributions were plotted for **(B)** germline V gene usage, **(C)** germline divergence, and **(D)** CDR3 loop length for heavy chains (left panel), κ chains (middle panel), and λ chains (right panel). Color coding denotes the 7 sequential time points with Day 4 shown in gray, Day 15 in red, Month 2 in orange, Month 3 in sky blue, Month 6 in green, Month 10 in purple, and Month 12 in black. The germline V genes used by ZK2B10 (IGHV1-8 and IGLV1-47) are marked with red dots.

Next-generation sequencing (NGS) is a powerful tool for probing antibody response to natural infection and vaccination (40–42). Extensive studies of broadly neutralizing antibodies (bNAbs) and their lineage development using NGS have revealed the unexpected complexity and diversity of B cell repertoire in HIV-1-infected individuals during chronic infection (37, 43–45). Here, we performed a longitudinal NGS analysis of the antibody repertoire in Pt1 to delineate the dynamic B cell response to ZIKV infection following the procedure outlined in Figure 1A. We analyzed seven sequential time points from the acute phase (Day 4 and Day 15 after the onset of symptoms) to the convalescent phase (Month 2, 3, 6, 10, and 12 after the onset of symptoms). We combined 5′-RACE PCR and single reverse primers in template preparation to ensure the NGS in a long-read (600 bp) and unbiased manner as previously reported (Figure S1) (34, 36, 46–48). Deep sequencing yielded a total of 14.2 million heavy chains and 14.1 million light (κ and λ) chains in two separate NGS runs on the Ion S5 GeneStudio platform (Table S1). The *Antibodyomics* 2.0 pipeline was used to process, annotate, and analyze the NGS data, rendering 1.3 to 2.9 million reads per time point (Table S1) (35, 49). Of these sequences, 55.3 to 71.2% are high-quality, full-length antibody variable regions which were used for the in-depth analysis of B cell repertoire profiles (Table S1). Furthermore, we traced the lineage of ZK2B10 within the NGS-derived repertoire and synthesized representative somatic variants for functional characterization both *in vitro* and *in vivo* (Figure 1A).

Overall, Pt1 exhibited a diverse and dynamic distribution of germline gene usage (Figure 1B). A few germline genes are dominant in all seven time points such as IGHV1-69, IGKV3-20, and IGLV1-40 with an average of 15.21% or greater (Figure 1B, left). In contrast, some specific germline genes were observed with low frequency, such as IGHV1-8, the V_H_ germline gene of ZK2B10, ranging from 0.98% to 4.80% in seven time points (Figure 1B, left). The V_L_ germline gene of ZK2B10, corresponding to IGLV1-47, ranged from 2.58 to 5.34% (Figure 1B, right). However, the low frequency of IGHV1-8 and IGLV1-47 was unexpected, suggesting that ZK2B10 did not represent a major B cell lineage in the repertoire spanning the acute and convalescent phases of ZIKV infection. In addition, there appeared to be no correlation between the potency of a ZIKV E-targeting mAb and its lineage expansion or prevalence, as indicated by the low frequency of the ZK2B10 germline gene family.

We then determined the degree of SHM, or germline divergence, at each time point from early acute phase to late convalescent phase. As shown in Figure 1C, there was a significant increase in the population of germline-like sequences at Day 15 for both heavy and light chains. As a result, the average SHM of heavy, κ and λ chain repertoires fell to 6.25, 5.92, and 5.91% at Day 15, respectively. Of note, the SHM decreased in most V gene families at Day 15, suggesting a repertoire-level response (Figure S2). As for the V_H_ germline gene of ZK2B10, IGHV1-8 showed only 6.45% SHM at Day 15 and varied between 7.23 and 13.60% at other time points (Figure S2, left). The V_L_ germline gene of ZK2B10, IGLV1-47, displayed 5.72% SHM at Day 15, and 6.70 to 9.04% at other time points studied (Figure S2, right). These results suggest a drastic shift in repertoire composition likely caused by a rapid plasmablast response during the acute phase of ZIKV infection. The emergence and development of ZK2B10 may serve as an example for this type of antibody response. These patterns are consistent with the fact that plasmablasts from ZIKV-infected, flavivirus-naïve individuals exhibited less somatic hypermutation or clonal expansion compared to those from ZIKV-infected, DENV-immune individuals, which may originate from common memory B cell clones (19, 50). Interestingly, similar patterns have also been reported for chronically infected HIV-1 patients in response to a rapidly evolving virus population (36).

Next, we determined the distribution of CDR3 loop length. Due to the diversity of the D gene, a rather dispersed distribution of HCDR3 loop length was observed as compared to a steady, canonical CDR3 loop length distribution obtained for κ and λ chains (Figure 1D). The HCDR3 loops were mainly distributed in the range of 9-aa to 15-aa (Figure 1D, left). As for the light chain, 9-aa KCDR3 loops accounted for 79.3 to 88.2% of the κ chain repertoire, while 9-aa to 11-aa LCDR3 loops accounted for 81.3 to 94.9% of the λ chain repertoire (Figure 1D, middle and right). Results from the case study of Pt1 have revealed unique features of human B cell repertoire during acute and transient ZIKV infection. Future studies with longitudinal samples from a larger cohort of infected donors would be needed to validate our findings.

### ZK2B10 Lineage-Specific Antibody Response During ZIKV Infection

**Figure 2:**
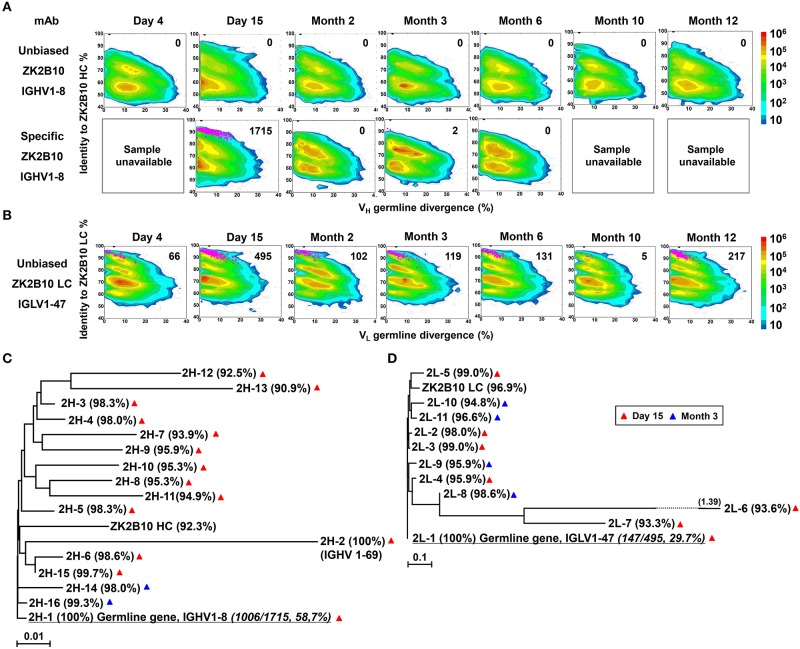
Lineage tracing of ZK2B10 in the Pt1 repertoire across ZIKV infection. **(A)** Lineage tracing of ZK2B10 heavy chain in unbiased antibody repertoire (upper panel) and in ZK2B10 gene-specific antibody population (lower panel). For ZK2B10 gene-specific sequence analysis, the PBMCs samples at Day 4, Month 10 and Month 12 were unavailable, which are marked as sample unavailable. **(B)** Lineage tracing of ZK2B10 λ chain in unbiased antibody repertoire. For each time point, the repertoire is shown as a two-dimensional (2D) plot, with the X-axis indicating the germline divergence of NGS-derived antibody sequences and the Y-axis for their identity with respect to ZK2B10 heavy or λ chain. Color coding indicates the sequence density on the 2D plots ranging from 10^1^ to 10^8^. ZK2B10 heavy and λ chains are shown as black dots on the 2D plots. CDR3-defined somatic variants that are evolutionarily related to ZK2B10 heavy and λ chain are shown as magenta dots, with the total number of reads labeled on the 2D plots (HC: heavy chain; LC: λ chain). **(C,D)** Neighbor-joining tree (MEGA6.0) depicting the relationship between the germline gene and representative ZK2B10 heavy **(C)** and λ **(D)** chain variants. Individual variants are labeled at the end nodes of each tree branch, alongside with their germline identity. Branch lengths are drawn to scale so that the genetic distance between different nucleotide sequences can be assessed. Color-coding of triangle indicates their emerging time during infection.

To probe the maturation pathway of ZK2B10, we traced the mAb lineage at each time point within the NGS-derived repertoire (Figures 2A,B). A CDR3 identity of 95% with respect to WT ZK2B10 heavy or λ chain was used as the cutoff for identifying sequences evolutionarily related to ZK2B10 (Figures 2A,B, shown as magenta dots on the 2D plots). As reported previously, WT ZK2B10 was derived from the memory B cells of Pt1 at Month 3 after the symptom onset (24). Unexpectedly, from the libraries of unbiasedly amplified germline gene families, we could not find any ZK2B10 heavy chain somatic variants in the repertoire at all seven time points, suggesting that the ZK2B10 lineage may have an extremely low frequency (Figure 2A, upper panel). To gain more insight into the ZK2B10 lineage evolution, we performed an additional NGS experiment on four antibody libraries at Day 15, Months 2, 3, and 6, using a degenerate forward primer to target the ZK2B10 heavy chain and its putative germline gene, IGHV1-8 (Figure 2A, lower panel). Gene-specific NGS yielded 1715 ZK2B10-like heavy chains for Day 15 and only two for Month 3 (Figure 2A, lower panel). As for λ chain repertoire, ZK2B10 λ chain somatic variants were detectable at Day 4 but reached the peak at Day 15 with 495 identified variants, and persisted into Month 12 despite a noticeable decline at Month 10 (Figure 2B). Of note, due to the lack of D gene segments, light chains do not possess unambiguous sequence signatures for CDR3-based lineage tracing. Nonetheless, our data suggests that ZK2B10 and its variants were induced rapidly and transiently at the end of acute phase during ZIKV infection. Interestingly, the majority of ZK2B10 somatic variants showed a germline divergence of <5.0% in both heavy and λ chain repertoires (Figure 2A, low panel and Figure 2B).

**Figure 3:**
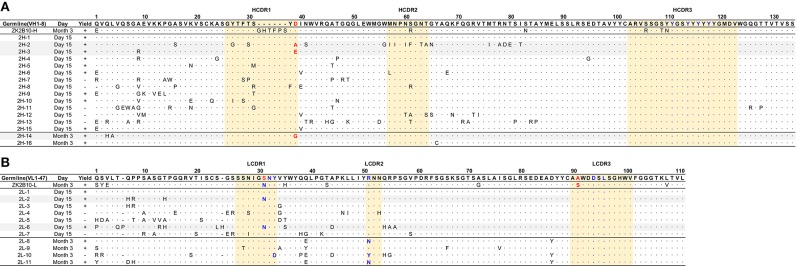
Sequence alignment of representative somatic variants of ZK2B10. **(A,B)** Sequence alignment of representative ZK2B10 heavy **(A)** and λ **(B)** chain variants with their CDR regions highlighted. Residues that directly bind to ZIKV E DIII are colored in blue according to the crystal structure of ZK2B10 in complex with ZIKV E DIII (31). The identified hotspot residues that potentially critical for ZK2B10 maturation are marked in red.

To further study the maturation pathway of ZK2B10, we selected representative somatic variants for antibody synthesis and functional characterization. A hierarchical clustering method was used for sequence selection, as previously described (34). In addition to the dominant sequences, a consensus selection was conducted based on the sequence characteristics to ensure broad coverage and representativeness (34). Of these, 16 representative heavy chains were selected with 14 from Day 15 and 2 from Month 3, and 11 representative λ chains with 7 from Day 15 and 4 from Month 3 (Figures 2C,D). These somatic variants were designated based on the order in which they were selected (e.g., 2H-1 is the 1st selected sequence of representative ZK2B10 heavy chain somatic variants). Surprisingly, 2H-1 and 2L-1 are 100% identical to their putative germline genes, corresponding to IGHV1-8 and IGLV1-47, with NGS read frequencies as high as 58.7% (1006/1715) and 29.7% (147/495), respectively (Figures 2C,D). Furthermore, all these ZK2B10 somatic variants showed a low degree of SHM: the average identity of representative heavy chains with respect to their putative germline gene, IGHV1-8, was 96.81%; as for λ chains, the average germline identity to IGLV1-47 was also as high as 96.79% (Figures 2C,D). Sequence alignment of variable regions of representative ZK2B10 somatic variants is shown in Figure 3. To summarize, the ZK2B10 antibody lineage represents a transient plasmablast response with a low degree of SHM at the end of acute phase of ZIKV infection.

### Functional Characterization of ZK2B10 Somatic Variants

**Figure 4:**
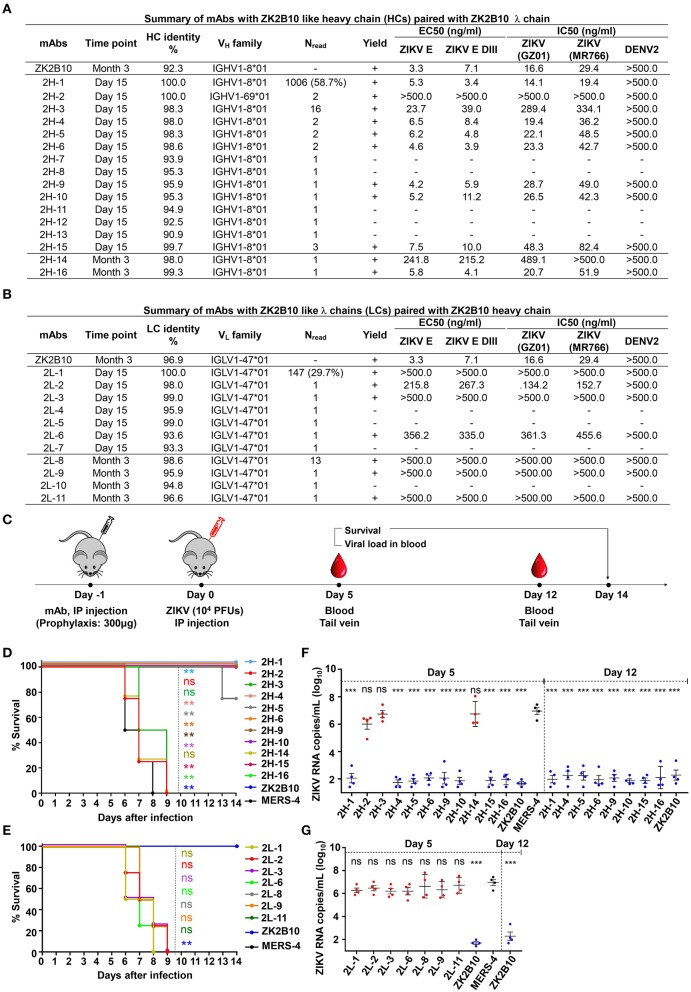
Summary of somatic variants of the ZK2B10 antibody lineage**. (A,B)** ZK2B10 somatic variants are listed with the sampling time point, genetic characterization, sequencing read frequency (N_read_), expression yield, and functional characterization. **(A)** 16 representative ZK2B10 heavy chain variants and **(B)** 11 representative ZK2B10 λ chain variants identified from the Day 15 and Month 3 antibody repertoires. EC_50_ represents the half-maximal effective concentrations for ELISA binding assays. IC_50_ represents the half-maximal inhibitory concentrations for plaque neutralization assays. **(C–G)** Antibody protection against lethal ZIKV challenge in AG6 mice. Shown here: **(C)** timeline for mAb injection, ZIKV inoculation, and blood collection. The prophylactic potential of mAbs was assessed by monitoring survival rates for representative **(D)** heavy and **(E)** λ chain somatic variants of ZK2B10 up to 14 days post challenge, and ZIKV RNA copies in blood for **(F)** heavy and **(G)** λ chain somatic variants of ZK2B10 on 5 days and 12 days post challenge. Single measurement of ZIKV RNA copies in blood showed statistically significant difference among study groups, each containing four animals. All data are presented here as mean ± SEM. ^*^p < 0.05; ^**^p < 0.01; ^***^p < 0.001; ns, not significant.

The representative ZK2B10 somatic variants were then synthesized and paired with their respective wild-type (WT) partner chains for full-length human IgG1 expression and functional characterization. Of the 16 synthesized ZK2B10 heavy chain somatic variants, 11 (2H-1, −2, −3, −4, −5, −6, −9, −10, −14, −15, and −16) could be expressed when paired with WT ZK2B10 λ chain (Figure 4A). We next measured their binding to E protein and E DIII of ZIKV by ELISA. A mong the 11 mAbs, 8 (2H-1, −4, −5, −6, −9, −10, −15, and −16) demonstrated strong binding affinities for E and E DIII at a similar level to ZK2B10, with the half-maximal effective concentrations (EC_50_) ranging from 3.4 to 11.2 ng/ml, while the remaining 3 mAbs (2H-2, −3, and −14) showed low affinities (Figure 4A). ZIKV E protein and E DIII were expressed in bacterial cells following the same procedure as previously described for the crystallographic analysis (31, 39). It must be noted that the affinity of a mAb may vary depending on the expression system used to produce the antigen. Nonetheless, the affinity of WT ZK2B10 here (EC_50_ = 3.3 ng/mL) was comparable to that obtained using HEK293T-derived ZIKV E (EC_50_ = 8.4 ng/mL), confirming the antigen binding of ZK2B10 somatic variants (24). These mAbs were then employed in plaque reduction neutralization tests against two ZIKV strains, GZ01 (Asian) and MR766 (African), and DENV 2 (Figure 4A). Consistent with their binding affinities, the 8 strong binders (2H-1, −4, −5, −6, −9, −10, −15, and −16) neutralized GZ01 and MR766 potently (Figure 4A). The half-maximal inhibitory concentrations (IC_50_) ranged from 14.1 to 82.4 ng/ml, which are comparable to WT ZK2B10 and other potent E-targeting mAbs isolated from ZIKV-infected, DENV-naïve human subjects (Figure 4A) (18, 20, 21, 24, 26). Not surprisingly, 2H-2 failed to show detectable potency (IC_50_ >500 ng/ml) against both GZ01 and MR766, while 2H-3 exhibited only modest neutralizing activity (IC_50_ = 289.4 ng/ml to GZ01 and 334.1 ng/ml to MR766). Similarly, 2H-14 showed negligible or no neutralizing activity against the two ZIKV strains tested (IC_50_ = 489.1 ng/ml to GZ01 and IC_50_ >500 ng/ml to MR766) (Figure 4A). All these mAbs showed no cross-neutralizing activity with DENV 2 (Figure 4A). Strikingly, with 100% identity to IGHV1-8, 2H-1 showed high affinity for full-length E and E DIII of ZIKV with EC_50_ values measured at 5.3 ng/ml and 3.4 ng/ml, respectively (Figure 4A). Consistently, 2H-1 also displayed potent neutralization against GZ01 and MR766, with IC_50_ values of 14.1 ng/ml and 19.4 ng/ml, respectively (b). Notably, 1006 out of 1715 (58.7%) ZK2B10-like heavy chains from Day 15 were identical to 2H-1, confirming that this germline-like mAb lineage emerged at the peak of plasmablast response. Based on the alignment with the IGHV1-8 germline sequence, the functional loss of 2H-2, −3, and −14 could potentially be explained by the mutation of D39 located toward the end of HCDR1 (Figure 3A). As for the 11 synthesized ZK2B10-like λ chains, 7 (2L-1, −2, −3, −6, −8, −9, and −11) were expressible when paired with WT ZK2B10 heavy chain. Of note, 5 of these 7 λ chain variants (2L-1, −3, −8, −9, and −11) failed to bind ZIKV E or E DIII and showed undetectable neutralization against ZIKV (Figure 4B). Among these λ chain variants, the sequence of 2L-1 is 100% identical to IGLV1-47 and represents a large portion of the Day 15 λ chains (147 out of 495, 29.7%) (Figure 4B). The reconstituted mAbs containing 2L-2 and 2L-6 demonstrated rather weak binding and neutralization compared to WT ZK2B10 (Figure 4B). These functional and repertoire observations, together with the sequence alignment, suggest that S31N on LCDR1 and A91S on LCDR3 could be critical for the maturation of ZK2B10 λ chain (Figure 3B).

Taken together, results from the functional characterization of ZK2B10 heavy chain somatic variants confirmed the hypothesis that this mAb lineage represents a transient yet effective naïve B cell response to ZIKV infection. The loss of function observed for most λ chain somatic variants, in which V_L_ was reverted to IGLV1-47, suggested that light chain maturation is crucial for the ZK2B10 lineage to acquire its potency and specificity, reminiscent of the HIV-1 bNAb, VRC01 (36).

### Protective Potential of ZK2B10 Somatic Variants in a Mouse Model

Previously, we have demonstrated that ZK2B10 can protect mice from lethal ZIKV infection and microcephaly (24, 30). Conducting similar animal studies will not only confirm the accuracy of our repertoire analysis but also provide useful clues as to the functional diversity of the ZK2B10 lineage *in vivo*. To this end, we tested the *in vivo* protection of representative ZK2B10 somatic variants against ZIKV lethal infection in AG6 mice (C57BL/6 mice deficient in IFNα, -β, and -γ receptors) following the protocol outlined in Figure 4C (24, 30, 51, 52). Briefly, we administered 300 μg of each ZK2B10-like mAb, ZK2B10 as positive control, or MERS-4 as negative control to groups of four AG6 mice, each 4–6 weeks in age, via the intraperitoneal (i.p.) route (Fig 4C) (38). On the following day, the animals were challenged with 10^4^ plaque-forming units (PFUs) of ZIKV Asian strain GZ01 via the intraperitoneal (i.p.) route (Figure 4C). Animals were monitored for survival rate up to 14 days after ZIKV challenge, and for viral RNA level in the blood on days 5 and 12 post ZIKV challenge (Figure 4C). As expected, *in vivo* protection of mAbs was correlated with their *in vitro* neutralization, as previously reported (30). For example, the heavy chain variants with potent neutralizing activities *in vitro* (2H-1,−4,−6,−9,−10,−15, and−16) provided complete protection with a survival rate of 100% up to 14 days after ZIKV challenge (Figure 4D). The RNA load in these groups was suppressed in blood with distinguishable level from the MERS-4 group (Figure 4F). Conversely, 2H-2,−3, and−14 failed to offer any protection with a median survival time of 7.25 to 8 days after ZIKV challenge (Figure 4D). The viral RNA levels measured in mice treated by these variants were on average 3.72–4.45 log_10_ greater than the ZK2B10 group at day 5 after ZIKV challenge (Figure 4F). In contrast, all λ chain variants demonstrated a consistent survival rate identical to that of the negative control MERS-4 and failed to suppress viral replication (Figures 4E,G). Therefore, *in vivo* evaluation of representative ZK2B10 somatic variants confirmed the differential effect of heavy and λ-light chains on antibody function, consistent with the *in vitro* characterization by ELISA and neutralization assays.

### Critical Residues for ZK2B10 Functional Maturation

**Figure 5:**
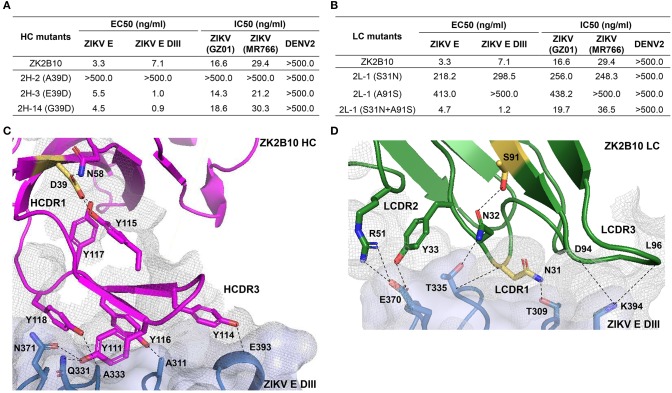
Mutagenesis and structural analyses of hotspot residues critical for ZK2B10 maturation. **(A)** Validation of ZK2B10 heavy chain critical residues by reverse mutagenesis. **(B)** Validation of ZK2B10 λ chain critical residues by mutagenesis. **(C)** Contact interface of ZK2B10 heavy chain (HC) with ZIKV E DIII. In the ribbon diagram, ZK2B10 HC is shown in magenta and ZIKV E DIII in cornflower blue. Residues directly involved in the contact interface with E DIII (Y111, Y114, Y116, and Y118) are shown with side chain. The identified critical residue, D39 on HCDR1, is highlighted in yellow. **(D)** Contact interface of ZK2B10 λ chain (LC) with ZIKV E DIII. ZK2B10 LC is shown in forest green and ZIKV E DIII in cornflower blue. Residues directly involved in the contact interface with E DIII (N31, N32, Y33, R51, D94, and L96) are shown with side chain. The identified critical residues, N31 on LCDR1 and S91 on LCDR3, are highlighted in yellow.

To further investigate the maturation pathway of the ZK2B10 lineage, we performed reverse mutagenesis and structural analysis to identify “hotspot” residues. Initially, we aligned the amino acid sequences of representative ZK2B10 heavy and λ chain variants with their putative germline genes, IGHV1-8 and IGLV1-47, respectively (Figure 3). For heavy chain variants, 2H-2, 2H-3, and 2H-14 lost their potency to ZIKV both *in vitro* and *in vivo*. These 3 heavy chains possess a single substitution mutation at residue D39 with respect to their germline gene (Figure 3A). To assess whether this was the cause of the reduced potency, we conducted reverse mutagenesis on 2H-2 (A39D), 2H-3 (E39D), and 2H-14 (G39D) and characterized the function of these mutants by ELISA and neutralization assays. As shown in Figure 5A, 2H-3 (E39D), and 2H-14 (G39D) mutants regained their ZIKV E-binding and neutralizing activities, approaching the level of WT ZK2B10. Due to the use of a different V_H_ germline gene, IGHV1-69, the 2H-2 (A39D) mutant was ineffective (Figure 5A). This result suggested that the ZK2B10 lineage has a restricted V_H_ gene usage to achieve high affinity and potency against ZIKV. Based on the crystal structure of ZK2B10 in complex with ZIKV E DIII, four residues within the HCDR3 loop (Y111, Y114, Y116, and Y118) are directly involved in the contact interface (31). Although D39 is within the HCDR1 loop, it forms hydrogen bonds with Y115 and Y117 on the opposite side of the HCDR3 loop, thus stabilizing the HCDR3 conformation (Figure 5C). These results provide further evidence that the ZK2B10 lineage was indeed generated during the naïve B cell response to acute ZIKV infection, with critical residues encoded by the germline gene. For λ chain variants, two critical mutations were identified that potentially contribute to the maturation of ZK2B10 lineage (Figure 3B). One such mutation, located on the LCDR1 loop, is N31, which is shared by two weakly functional variants, 2L-2 and 2L-6, as well as WT ZK2B10 λ chain (Figure 3B). The other mutation is at position 91 on LCDR3 loop, which is S91 in WT ZK2B10 λ chain but predominantly A91 in all weakly or non-functional λ chain variants (Figure 3B). Thus, N31 and S91 could potentially be the most critical mutations for ZK2B10 λ chain maturation. We first examined the effect of these two mutations individually by performing site-directed mutagenesis on 2L-1, which is 100% identical to germline gene IGLV1-47. Neither S31N nor A91S could render the germline antibody functional (Figure 5B). We then introduced a double mutation (S31N+A91S) into 2L-1, which, as expected, bound to ZIKV E and E DIII with high affinity and potently neutralized ZIKV at the same level of WT ZK2B10 (Figure 5B). As shown by the crystal structure, five residues within ZK2B10 λ chain (N31, N32, Y33, R51, D94, and L96) are directly involved in the contact interface (31). For the two NGS-identified hotspot residues, N31 directly interacts with T309 on ZIKV E DIII, while S91 forms a hydrogen bond with N32, which interacts with T335 on ZIKV E DIII (Figure 5D). In brief, our combined analysis of NGS data, antibody function, and complex structure confirms that residues N31 and S91 within the λ chain are essential to ZK2B10, thus representing a crucial event necessary for the functional maturation of this IGLV1-47-originated ZIKV E DIII-directed antibody lineage.

## Discussion

In this study, we delineated the B cell repertoire response of a ZIKV-infected individual (Pt1) during natural infection using an NGS-based approach. Future studies with sequential samples from more patients would be needed to validate and support our current findings. Our analysis showed an antibody repertoire profile with diverse germline usage, limited somatic hypermutation in variable genes, and steady CDR3 loop length. Tracing ZK2B10 in the NGS-derived antibody repertoire revealed the dynamics of an effective germline-encoded antibody lineage, which emerged prior to the convalescent phase of ZIKV infection. Germline-like somatic variants derived from the ZK2B10 lineage potently neutralized ZIKV and protected mice from lethal ZIKV challenge, while showing no cross-reactivity with DENV 2. We also demonstrated that two mutations, N31 and S91, within the germline-encoded λ-light chain are essential to the functional maturation of this IGHV1-8/IGLV1-47-encoded antibody lineage.

Two important aspects in this study are worth highlighting. One is the effective germline-encoded antibody response represented by the ZK2B10 lineage. We observed a significant increase in germline-like antibodies at Day 15 after the onset of symptoms. This drastic shift in repertoire composition was likely a result of rapid plasmablast response toward the end of the acute phase of ZIKV infection. Interestingly, this shift coincided with the emergence of the ZK2B10 lineage, which exemplifies the role of the germline-encoded antibody response during ZIKV infection. Similar patterns have been described in previous studies. For monoclonal antibodies, germline-like human mAbs m301 and m302 were reported that target ZIKV E DIII cryptic epitopes (C-C' loop) and neutralize ZIKV potently both *in vitro* and *in vivo* (22). Another human mAb, P1F12, originates from germline gene IGHV3-7 with an identity of 100% and neutralizes ZIKV potently as well (53). For the overall B cell response, plasmablast-derived antibodies from a ZIKV-infected, DENV-naïve donor showed low levels of SHM, supporting the mechanism of naïve B cell activation (19). Low levels of IgG SHM were also reported during acute DENV infection, which is consistent with an “innate-like” antiviral recognition mediated by B cells possessing antigen-specific naïve B cell receptors (54). Therefore, the induction of germline-encoded neutralizing antibodies is critical for effective protection against acute flavivirus infection. Diverse mechanisms of antibody lineage development have been found in chronic infections. During HIV-1 infection, rapidly emerged MPER-directed antibody lineages have been reported to achieve neutralizing breadth with low levels of SHM (55). In contrast, HIV-1 bNAbs VRC01 and PGT121, which target the CD4 binding site (CD4bs) and the V3 stem, respectively, require extensive mutation to achieve neutralizing breadth and potency (34, 36). In addition, long HCDR3 loops are required for bNAb PGT121 to penetrate the glycan shield of the envelope spike. In summary, our longitudinal analysis of Pt1 repertoire and ZK2B10 lineage development provides insight into the possible protective immunity against ZIKV infection. It must be noted that more ZIKV-infected donors need to be analyzed in future studies to confirm the B-cell repertoire dynamics in response to ZIKV infection observed for Pt1. However, longitudinal patient samples covering the entire course of infection are usually scarce due to the difficulties in early diagnosis and sample collection, posing a significant challenge for such studies.

The other important aspect of our study is its implication for rational design of a safe and effective ZIKV vaccine. As previously reported, ZK2B10 and other E DIII-specific mAbs, are ZIKV-specific, potently neutralizing, and can protect mice from a lethal ZIKV challenge (18, 23, 24, 30). Structural studies revealed that ZK2B10 binds to the residues within the lateral ridge of DIII and blocks infection at a post-attachment step similar to other E DIII-specific potently neutralizing mAbs (31, 50). In addition, E DIII-specific antibodies are critical for controlling ZIKV as they correlate positively with high neutralization titers and their depletion results in reduced neutralizing activity in ZIKV-infected patient serum (24, 50). Our results indicate that only two mutations of the IGLV1-47 germline λ chain, N31 and S91, can sufficiently enable the IGHV1-8/IGLV1-47 germline antibodies to achieve potent ZIKV neutralization. Therefore, this barrier could be readily overcome by an antigen-activated B cell repertoire. The low degree of SHM observed for the ZK2B10 lineage suggests that elicitation of naïve protective B cell response against ZIKV may be achieved with a standard vaccination regimen. Furthermore, E DIII-based vaccine has been reported to avert lethal West Nile virus (WNV) infection without enhancing ZIKV or DENV infectivity (56). However, the low frequency and transient expansion of ZK2B10-like antibodies observed in the Pt1 repertoire suggest that overcoming the suboptimal immunogenicity of ZIKV E DIII, an elongated immunoglobulin-like domain, may prove to be a significant challenge for ZIKV vaccine development (39, 57). While many E DIII-directed antibodies are potent neutralizers, antibodies targeting the quaternary epitopes have also been reported with exceptional neutralizing potency (13, 28, 58). Longitudinal analysis of such quaternary antibodies will provide valuable insight into the protective immunity against ZIKV infection and inform rational vaccine design, warranting further investigations.

## Data Availability Statement

The datasets analyzed in this manuscript are not publicly available. Requests to access the datasets should be directed to jiang@scripps.edu.

## Ethics Statement

The studies involving human participants were reviewed and approved by Ethical Committee of the Guangzhou Eighth People's Hospital, Guangzhou Medical University. The patients/participants provided their written informed consent to participate in this study. The animal study was reviewed and approved by Experimental Animal Welfare and Ethics Committee of Tsinghua University.

## Author Contributions

Project design by FG, XL, LZ, JZ, and LY. Sample preparation by FG, XS, FZ, CY, and LZ. Library preparation and NGS by LH. Data processing and annotation by XL and JZ. Antibody lineage tracing by XL and JZ. Antibody sequence selection by FG, XL, and JZ. Antibody synthesis by FG, RW, and LZ. Antigen binding and neutralization assays by FG and LZ. ZIKV challenge and protection in mice by FG, HW, and LZ. Manuscript written by FG, LZ, JZ, and LY.

### Conflict of Interest

The authors declare that the research was conducted in the absence of any commercial or financial relationships that could be construed as a potential conflict of interest.
