# Supplementary material for: Development of a Potent and Protective Germline-Like Antibody Lineage Against Zika Virus in a Convalescent Human
Source: Front Immunol. 2019 Oct 24;10:2424. doi: 10.3389/fimmu.2019.02424 (PMC6821881; doi:10.3389/fimmu.2019.02424)
Supplement: Supplementary file 1 [file Table_1.pdf]

S1 Table. Next-generation sequencing (NGS) of antibody repertoires of a ZIKV-infected Chinese patient.

A. Unbiased heavy and light chain repertoires obtained from 5'-RACE PCR and long-read NGS

Time point	N_{read}	Chain	N_{chain}	<Length> (nt)	Usable (%)
	2,251,803 (H)	H	1,778,286	577.3	1,168,643 (65.7%)
Day 4	2,573,704 (κ+λ)	κ	847,134	581.4	477,634 (56.4%)
	—	λ	1,129,678	616.0	684,407 (60.6%)
	2,903,255 (H)	H	2,243,427	576.4	1,466,278 (65.4%)
Day 15	2,422,687 (κ+λ)	κ	945,850	613.4	526,669 (55.7%)
	—	λ	1,002,926	613.9	618,428 (61.7%)
	1,520,713 (H)	H	1,301,551	586.7	907,084 (69.7%)
Month 2	1,893,984 (κ+λ)	κ	740,365	606.0	455,337 (61.5%)
	—	λ	858,739	625.6	540,325 (62.9%)
	1,810,255 (H)	H	1,505,394	565.4	1,008,904 (67.0%)
Month 3	1,902,224 (κ+λ)	κ	686,624	588.9	393,331 (57.3%)
	—	λ	829,831	614.9	512,005 (61.7%)
	2,035,707 (H)	H	1,607,945	576.9	1,072,581 (66.7%)
Month 6	1,838,536 (κ+λ)	κ	750,002	604.5	414,641 (55.3%)
	—	λ	746,624	617.2	475,717 (63.7%)
	1,452,961 (H)	H	1,292,264	576.8	919,669 (71.2%)
Month 10	1,356,757 (κ+λ)	κ	484,221	604.1	306,232 (63.2%)
	—	λ	747,315	614.0	515,400 (69.0%)
	2,243,832 (H)	H	1,722,355	560.6	1,121,665 (65.1%)
Month 12	2,114,917 (κ+λ)	κ	838,478	607.7	473,380 (56.5%)
	—	λ	911,970	622.4	564,912 (61.9%)
B. ZK2B10 heavy chain repertoires obtained from gene-specific PCR and long-read NGS					
Day 15	2,903,024	H	2,368,840	431.9	1,690,019 (71.3%)
Month 2	3,526,417	H	2,688,196	428.9	1,791,398 (66.6%)
Month 3	4,200,005	H	3,278,646	426.5	2,303,157 (70.2%)
Month 6	3,696,344	H	2,909,350	427.5	2,022,506 (69.5%)

[a] Antibody library preparation was performed using previously reported PCR procedures, namely 5'-RACE PCR for unbiased repertoire analysis and gene-specific PCR for ZK2B10 lineage analysis. Human IgG/IgM reverse primers were used for library preparation, with sequencing performed on the Ion GeneStudio S5 platform using a 530 chip. Listed items include time point, total number of raw reads (heavy and light chains libraries were sequenced on two separate 530 chips), antibody chain type, number of confirmed antibody chains after germline gene assignment with a cutoff E-value of 10^{-3}, average read length, and percentage of usable sequences after *Antibodyomics* pipeline processing and filtering with a cutoff of 250bp for variable gene alignment.
