# Supplementary figures and images for: Development of a Potent and Protective Germline-Like Antibody Lineage Against Zika Virus in a Convalescent Human
Source: Front Immunol. 2019 Oct 24;10:2424. doi: 10.3389/fimmu.2019.02424 (PMC6821881; doi:10.3389/fimmu.2019.02424)

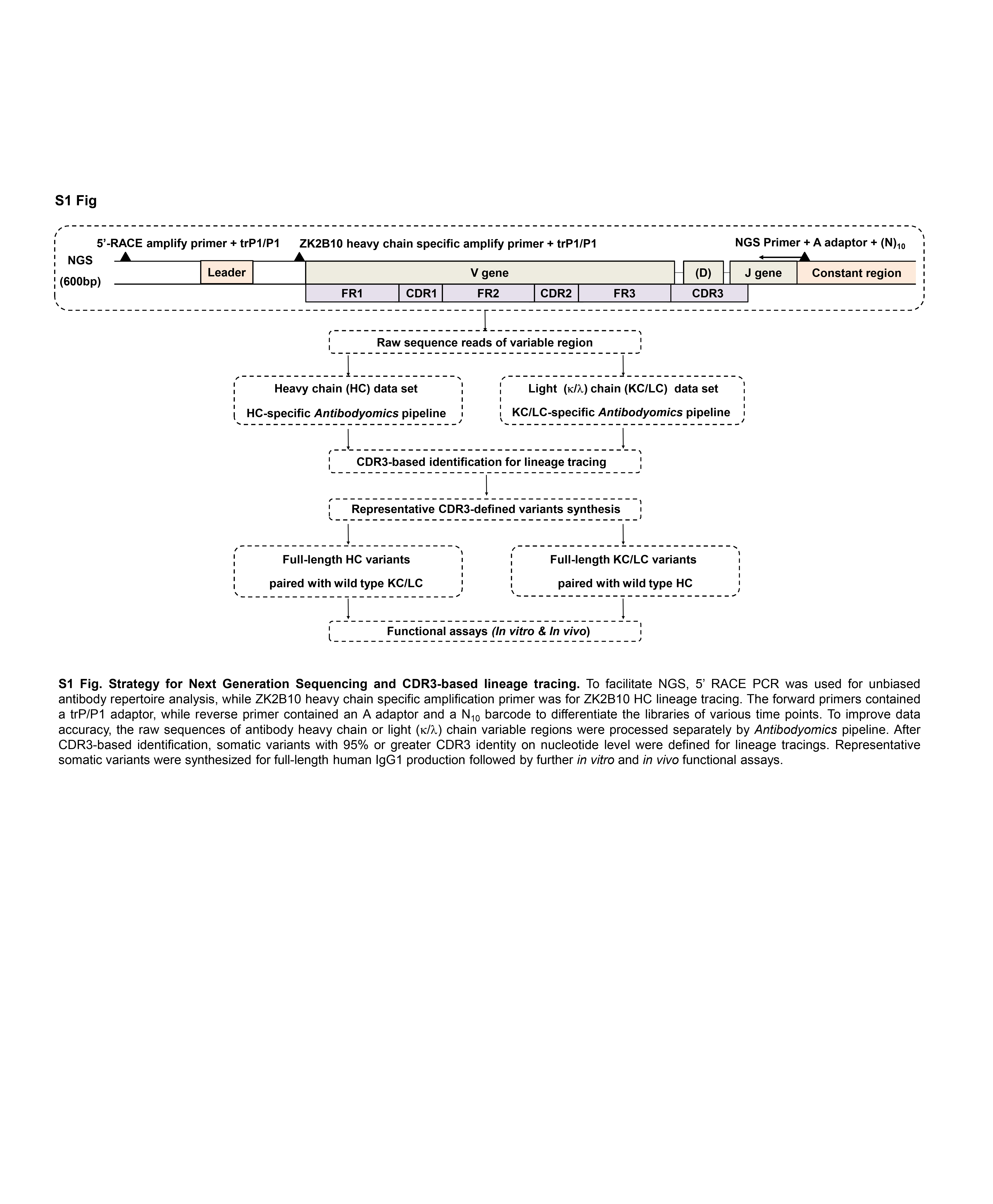

Supplement: Supplementary file 2 [file Image_1.jpg]

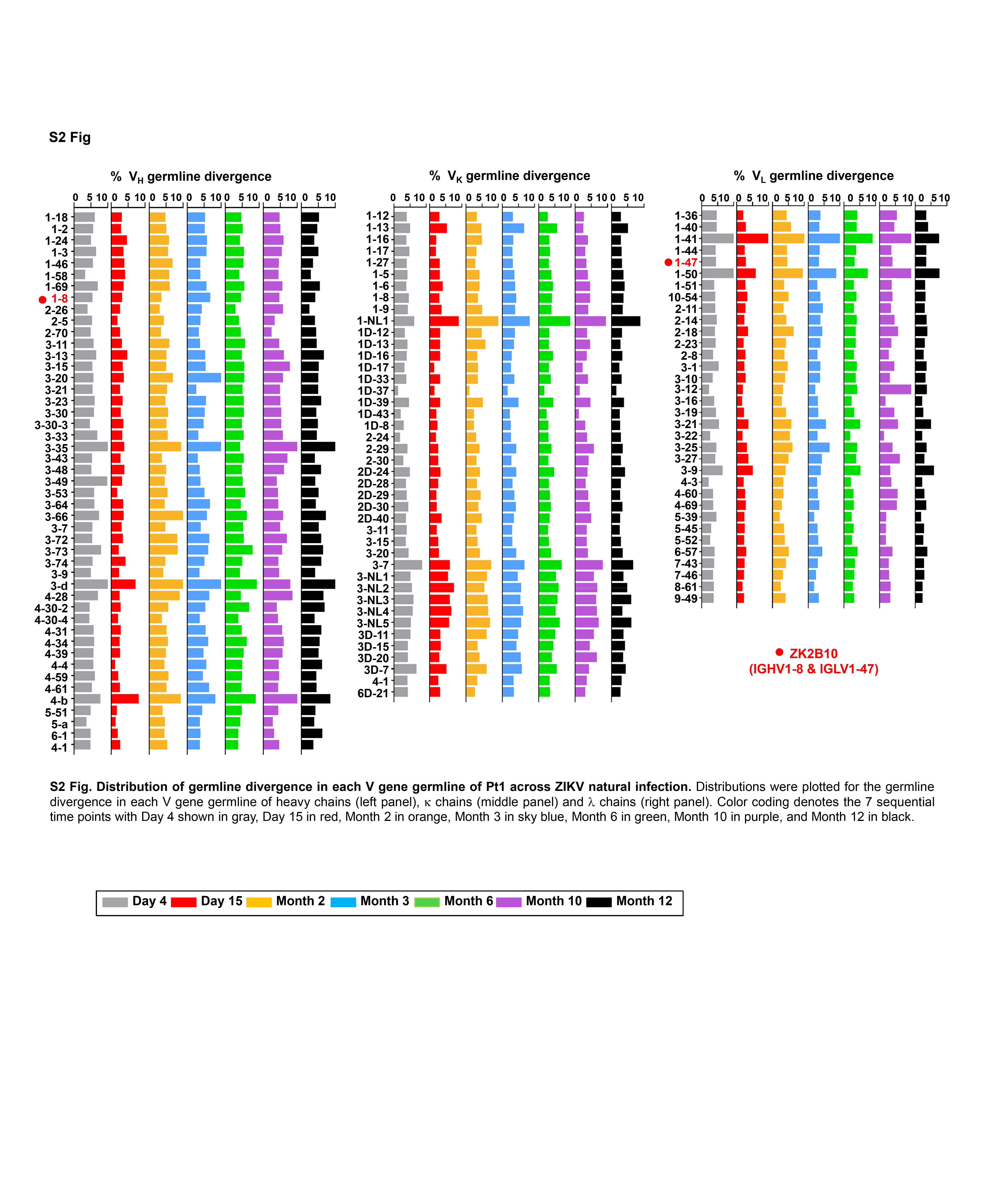

Supplement: Supplementary file 3 [file Image_2.jpg]
